# Supplementary material for: Efficacy and Safety of Auricular Acupuncture for the Treatment of Insomnia in Breast Cancer Survivors: A Randomized Controlled Trial
Source: Cancers (Basel). 2021 Aug 13;13(16):4082. doi: 10.3390/cancers13164082 (PMC8394534; doi:10.3390/cancers13164082)
Supplement: Supplementary file 1 [file cancers-13-04082-s001.zip › cancers-1277180-supplementary.pdf]

Table S1: Auricular acupuncture protocol of each patient and primary outcome

	**Auricular acupuncture point**																		
				Concha-zones							psychotropic points								
Patient ID	Postanti-tragal belt	helix channel	Sciatic nerve	heart	lung	spleen	liver	kidney	bladder	(tragus) adrenal	1	2	3	4	sun	Vegetative point II	sympathetic	Shen men	Change PSQI Week 0–5
01	x	x					x					x						x	−9
03	x	x				x		x				x						x	−8
05	x	x						x		x		x						x	−9
08	x	x					x	x				x						x	−6
09	x	x		x			x					x						x	0
11	x	x					x			x		x					x	x	−7
15	x	x					x		x	x							x	x	0
16	x	x					x			x			x					x	−9
18	x	x						x		x		x						x	−6
20	x	x			x		x			x								x	−6
22	x	x					x	x		x		x						x	−8
24	x	x	x				x									x		x	−2
26	x	x					x	x		x								x	−3
28	x	x					x			x		x						x	−7.2
30	x	x			x					x		x						x	1
32	x	x					x	x				x						x	−9
34	x	x		x			x	x		x		x						x	−1
37	x	x		x			x							x			x	x	−5
38	x	x		x			x								x			x	−2
40	x			x			x			x							x	x	−1
42	x	x		x			x						x					x	0
44	x	x			x				x	x					x			x	3
45	x	x					x	x		x					x			x	−3
48	x	x					x	x		x		x						x	−4.5

49	x	x		x			x	x		x			x					x	−5
52	x	x					x		x	x		x						x	−6
Abbreviation: PSQI: Pittsburgh Sleep Quality Index.																			
